# Physicochemical Position-Dependent Properties in the Protein Secondary Structures

**DOI:** 10.29252/.23.4.253

**Published:** 2019-07

**Authors:** Ehsan Saghapour, Mohammadreza Sehhati

**Affiliations:** 1 Department of Bioelectronic and Biomedical Engineering, School of Advanced Technologies in Medicine, Isfahan University of Medical Sciences, Isfahan, Iran; 2 Medical Image and Signal Processing Research Center, Isfahan University of Medical Sciences, Isfahan, Iran

**Keywords:** Algorithms, Amino acids, Physicochemical, Protein structure

## Abstract

**Background:**

Establishing theories for designing arbitrary protein structures is complicated and depends on understanding the principles for protein folding, which is affected by applied features. Computer algorithms can reach high precision and stability in computationally designed enzymes and binders by applying informative features obtained from natural structures.

**Methods:**

In this study, a position-specific analysis of secondary structures (α-helix, β-strand, and tight turn) was performed to reveal novel features for protein structure prediction and protein design.

**Results:**

Our results showed that the secondary structures in the N-termini region tend to be more compact than C-termini. Decoying periodicity in length and distribution of amino acids in α-helices is deciphered using the curve-fitting methods. Compared with α-helix, β-strands do not show distinct periodicities in length. Also, significant differences in position-dependent distribution of physicochemical properties are shown in secondary structures.

**Conclusion:**

Position-specific propensities in our study underline valuable parameters that could be used by researchers in the field of structural biology, particularly protein design through site-directed mutagenesis.

## INTRODUCTION

Understanding the relationship between position-specific properties of amino acids sequence and the secondary structure formation is vital for protein structure prediction and *de novo* protein design. The first ideas of protein structure prediction and *de novo* protein design come from very early studies on the correlation of amino acid distribution in protein structures[1-4]. It has been shown that the occurrence of amino acids in local structures, e.g. secondary structure, is position-dependent[5-9]. Recent studies have explored more details of amino acid distribution in secondary structures[5,10-15] and their functional roles[16,17]. In addition, the physicochemical bases that dictate the preference or avoidance of the amino acids for the secondary structure formation have been reported in a number of investigations[18,19]. These properties would be useful for designing algorithms to encode the molecular structures of natural proteins, which would improve the stability and precision of the resulting proteins[20]. However, the lack of comprehensive studies on position-specific evolutionary conservation and physicochemical properties of amino acids in secondary structures have motivated us to investigate these matters in the current research work.

In this study, to extract rules governing position-specific preference or avoidance of amino acids in secondary structures, an extensive analysis was performed based on position-specific distribution and conservation of amino acids in secondary structures, as well as based on the position-specific physicochemical properties of amino acids in secondary structures. This analysis was conducted on a database of secondary structure segments, including helical segments, β-strands, and tight turns (δ-turns, γ-turns, β-turns, α-turns, and π-turns). Our result introduces novel rules that govern formation and stabilization of secondary structures.

## MATERIALS AND METHODS

### Utilized dataset

The PDB database was culled at 25% sequence identity by PISCES webserver[21]. The structures of the selected proteins were determined via X-ray crystallography with resolution higher than 2 Å and R-factor value lower than 0.3. The sequences were excluded for proteins smaller than 40 amino acids. Additionally, we discarded the PDB files containing protein chains with chain break(s) and/or high frequency of nonstandard residues. This attempt resulted in a database containing 5362 non-redundant protein chains, corresponding to a total number of 1,197,533 amino acid residues. We used the standard method of definition of secondary structure of proteins to derive the secondary structure information from the remaining PDB files[22]. Using this database, the secondary structure information for α-helix (H), β-strand (E), and tight turn (T) were selected for further investigation. We separated tight turns based on their classification into subclasses δ-turn, γ-turn, β-turn, α-turn, and π-turn[23]. Consequently, three main subsets were formed with 34422, 63279, and 53192 sequences corresponding to α-helix, β-strand, and tight turn, respectively.

### Definition of specific positions in secondary structures

**Fig. 1:**
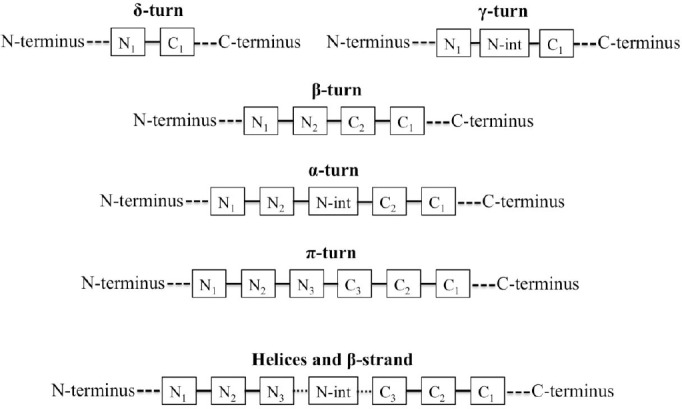
A schematic representation of amino acid positioning in different secondary structures considered in this study. int, intermediate

Amino acid positions in secondary structures were annotated as N*_i_* and C*_i_*, where N and C are the N-terminus and C-terminus of the secondary structure, respectively, and *i* is the position number of the amino acid with respect to distance from reference terminus, i.e. N and C. For helices and strands, N-cap and C-cap are referred to the first residue that precedes and succeeds the helix or strand, respectively; both residues do not participate in the conformation[13,24,25]. The notation used for different secondary structures is illustrated in Figure 1.

### Amino acid propensities in the secondary structure elements

In order to investigate the relationship between a specific position in a secondary structure and the amino acid residue located at this position, we defined position-specific propensity 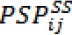 as follows:


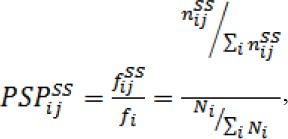


where 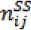 and 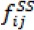 are the frequency and fraction of a given amino acid residue (type *i*) in position *j* of secondary structure, respectively. Also, *N_i_* and *f_i_* are the frequency and fraction of a given amino acid (type *i*) over the entire database.

### Position-specific physicochemical properties of amino acids

Up to now, the majority of statistical studies of position-specific secondary structure properties have been focused on distribution of amino acid residues in the secondary structure[10,12,26,27]. In this study, we analyzed diverse evolutionary and physicochemical properties of amino acid residues in the secondary structure elements including conservation, compactness, planarity of side chains, crystal contact, B-factor, and surface accessibility. Most of these features were extracted from PDBFIND2 (ftp://ftp.cmbi.ru.nl/pub/molbio/data/pdbfinder2/), using in-house written programs.

### Approximation of appropriate function for the obtained data

The Curve Fitting Toolbox of MATLAB V7.14 (R2012a) was applied to fit our data to smooth equations.

## RESULTS AND DISCUSSION

In this study, the provided database includes the sample size larger than that found in the literature such as Bhattacharjee and Biswas’ work[10], with only 2586 non-redundant protein chains. Also, as illustrated in Figure 1, a more comprehensive analysis was performed on all the secondary structures, including helical segments, β-strands, and tight turns (δ-turns, γ-turns, β-turns, α-turns, and π-turns) in contrast to the few limited structures that were considered in the similar studies[10-15].

### α-helices

Our database includes a large number of α-helices (n = 34422). The size of this database supports the reliable analysis of length distribution of α-helices and position-specific distribution of amino acids and physicochemical propensities in α-helices.

### Decaying periodicity in distribution of α-helices length

**Fig. 2:**
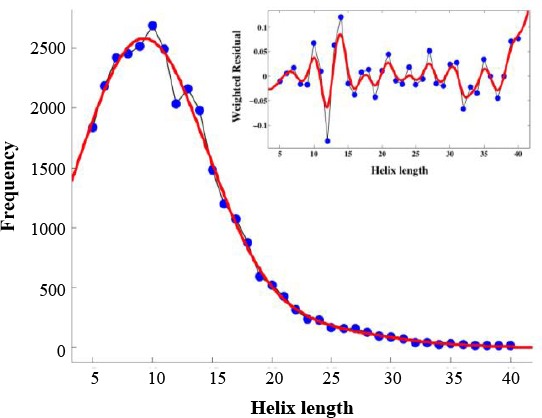
The distribution of helix length in the non-redundant protein database. Periodicity with a period corresponds to a ~3.6 residue repeat is apparent. The inset plot of weighted residuals demonstrates a dramatic preference for certain helical lengths Data are fitted to the equation: 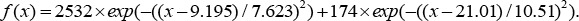

The number of different lengths of α-helices that observed in our database is illustrated in Figure 2. Only α-helices shorter than 20 amino acids occur more than 500 times. Besides, α-helices with 10 amino acids length are the most frequent. The number of residues in each helix is fitted to a smooth equation and supports previous reports[13,28]. We found that a vertically shifted Gaussian with two terms gave a much better fit than that reported earlier (R^2^ = 0.9953)[13,28].

The weighted residuals, as shown in the inset of Figure 2, confirm preference for certain helical lengths reported by other works[13,28]. In agreement with those works, the preferences are periodic, showing ~3.6 residue periodicity. However, the weighted residuals in Figure 2 highlighted a neat two-sided decaying periodicity in the region of α-helices shorter than 20 amino acids length, which cover over 70% of our large database. The weighted residuals defined in terms of the observed frequency *f_i_(O)* and the Gaussian fit *f_i_(P)* are as follows: *WR_i_=(f_i_(O)-f_i_(P))/f_i_(P)*.

### Periodicity in position-specific propensities of amino acids in α-helices

**Fig. 3:**
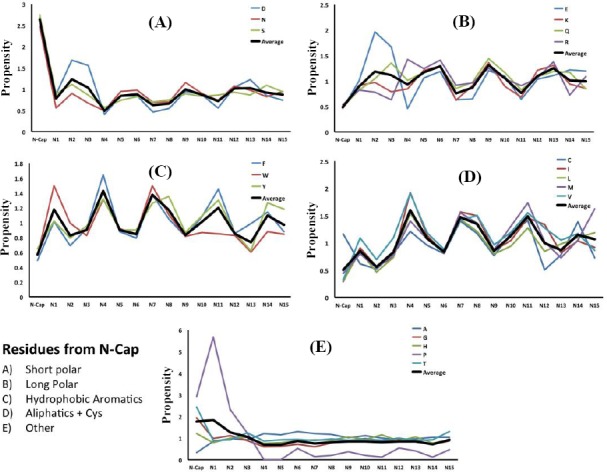
Position-specific propensities for single amino acids and amino acids in different physicochemical groups in the first 15 residues located at the N-terminus of helices in different categories.

**Fig. 4:**
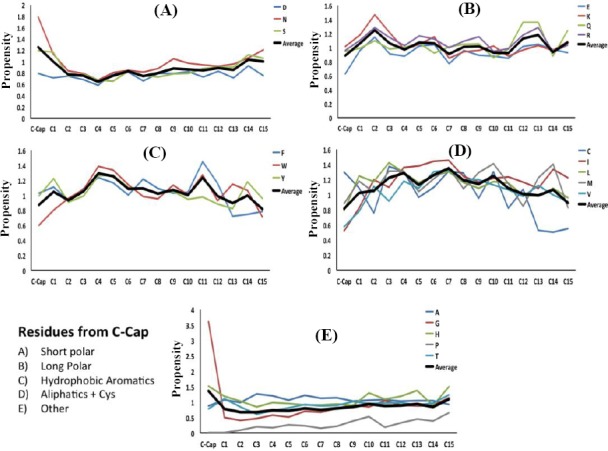
Position-specific propensities for single amino acids and amino acids in different physicochemical groups in the first 15 residues located at the C-terminus of helices in different categories.

**Fig. 5:**
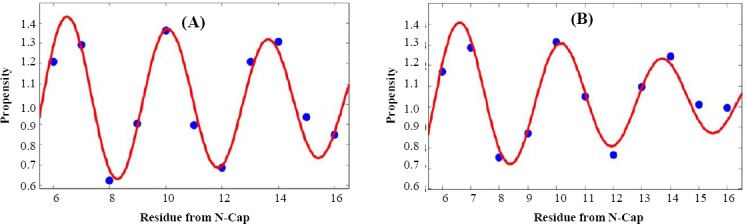
Position-specific amino acid propensities (blue circles) fitted to decaying sinusoid equation (red curve). The data for the residue Lysine (A) and for long polar amino acid residues (B) in positions 6–16 prior to the N-terminus are fitted to a decaying sinusoid equation (R^2^ = 0.94 and R^2^ = 0.95, respectively).

Figures 3 and 4 show the average propensities within helices for each amino acid, grouped based on the physicochemical properties of the amino acid. We examined position-specific propensities for the first 15 positions at both N-cap and C-cap in α-helices. As depicted in these Figures, we grouped the amino acids into five categories, including short polar (Figs. 3A and 4A, D, N, S), long polar (Figs. 3B and 4B, E, K, Q, R), aromatic (Figs. 3C and 4C, F, W, Y), hydrophobic aliphatic, and Cys (Figs. 3D and 4D, C, I, L, V, M), and other residues that do not fall into any one of these categories (Figs. 3E and 4E, G, H, P, T, A). Our results, as demonstrated in Figures 3 and 4, showed position-specific independency and periodicity of the presence of amino acids in helix. Besides, in a few cases, the data for positions 6-16 were fitted to a decaying sinusoid with R^2^ values over 0.9 (e.g. Fig. 5).

### Position-specific physicochemical propensities in α-helices

**Fig. 6:**
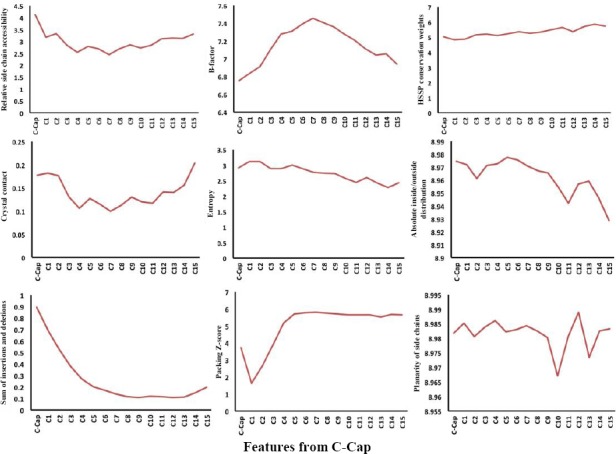
Position-specific physicochemical propensities in the first 15 residues located at the C-terminus of helices.

**Fig. 7:**
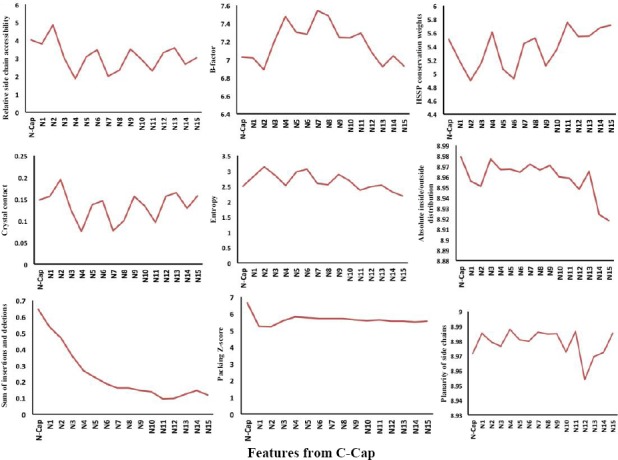
Position-specific physicochemical propensities in the first 15 residues located at the N-terminus of helices.

In addition to sequence-based position-specific propensities, we have analyzed position-specific physicochemical properties, including relative side chain accessibility, B-factor, conservation, crystal contact, entropy, absolute inside/outside distribution, insertions and deletions, packing, and planarity of side chains for the first 15 positions at both N-cap and C-cap in α-helices (Figs. 6 and 7). The comparison of the curves demonstrates a periodic pattern of residue positioning regarding their physicochemical properties This pattern is particularly observed for the 15 N-terminal residues within each helical conformation. Interestingly, the central positions of α-helices are highly conserved, and the N-terminus of α-helices is more compact compared to the C-terminus.

### β-strands

Length distribution, position-dependent distribution of amino acids, and physicochemical propensities in β-strands were studied for 63279 β-strand in our database. The obtained results confirm diversity in rules in formation and stabilization of the secondary structures.

### No periodicity in the distribution of β-strands length

**Fig. 8:**
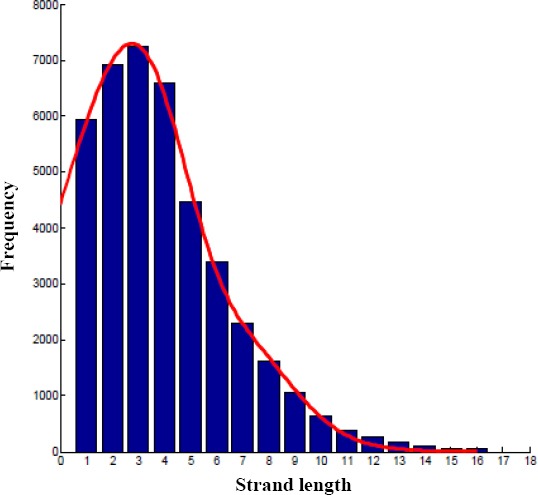
The distribution of β-strand length in the non-redundant protein database.

A plot of occurrence of strands, as the function of the strand length in our database, is illustrated in Figure 8. In our database, strands with five amino acids length are the most frequent; the result is consistent with previous reports[10,29]. The occurrence level decreased sharply for *β*-strands longer than six residues.

### Position-specific propensities of amino acids in β-strands

**Fig. 9:**
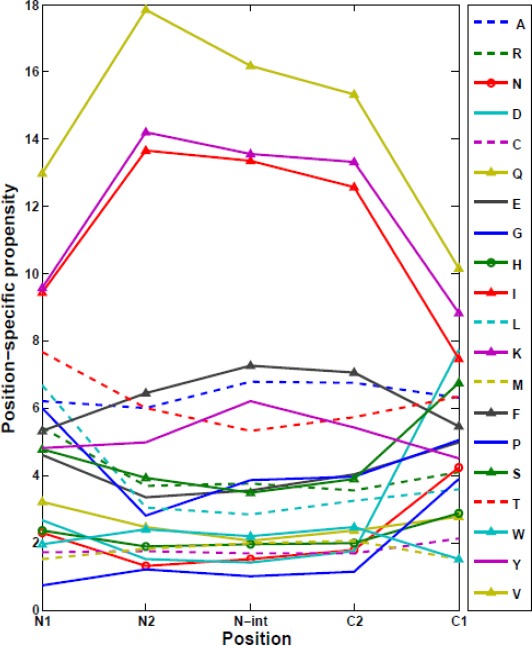
Position-specific amino acid propensities in five selected positions in β-strands.

Except for both N1 and C1 positions in β-strands, the average propensities of amino acid residues in other positions show no significant fluctuation. Figure 9 shows the average propensities of amino acid residues in five selected positions, i.e. N1, N2, N-int, C1, and C2. From our results, three amino acids, including glutamine, lysine, and isoleucine demonstrated higher local and global propensity in β-strands. Also, these three amino acids avoided in N1 and C1 positions. Interestingly, some amino acid residues preferred in one or both N1 and C1 positions. For instance, leucine preferred in N1 position but not in other positions.

### Position-specific physicochemical propensities in β-strands

Position-specific physicochemical properties for five different positions in β-strands, i.e. N1, N2, N-int, C1, and C2, were analyzed (Table 1). We observed that the central positions of β-strands have less insertions and deletions and the relative side chain accessibility, similar to our obtained results for α-helices. In addition, entropy constantly decreased from N- to C- termini. Interestingly, packing and B-factor show higher values in the middle and lower values on both N1 and C1 positions. However, packing in C1 position is slightly lower than N1 position. As we mentioned that the distribution of amino acids in C1 and N1 is different in β-strands (Fig. 9); therefore, the difference in compactness between the termini is indeed encoded in the primary sequence. Contrary to recent works that have evaluated a limited list of properties (propensity, χ^2^-values, hydrophobicity, and free energy) in β-strands[10,24], Table 1 provides a more complete picture of the secondary structures using physicochemical position-dependent properties in β-strands.

**Table 1:**
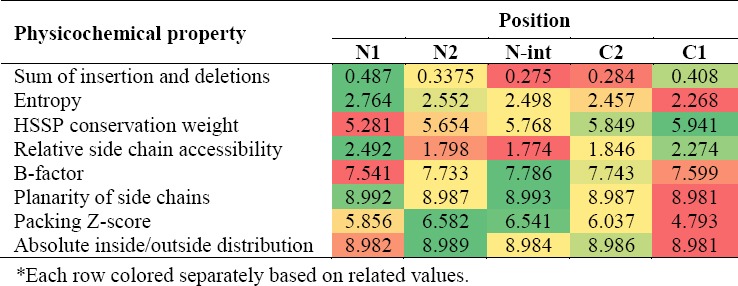
Physicochemical properties in β-strands

### Tight turns

Physicochemical features and Position-specific propensities of amino acids were calculated for tight turns, including δ-, γ- β-, α-, and π-turns, also called as 2-, 3-, 4-, 5-, and 6-turns, respectively (Tables 2 and 3). The obtained results demonstrated significant differences in position-dependent distribution of amino acid residues and physicochemical properties in tight turns. Pattern of preference of physicochemical properties was completely different in tight turn subclasses. However, packing in C1 position was slightly lower compared to N1 position in β-, α-, and π-turns (Table 2). Significant differences observed in the distribution of amino acids confirm that the differences in compactness between the termini are indeed encoded in the primary sequence.

**Table 2:**
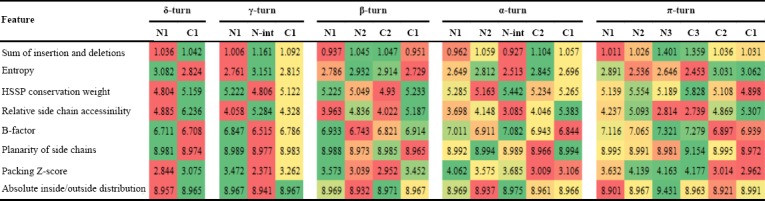
Physicochemical properties in tight turns

**Table 3:**
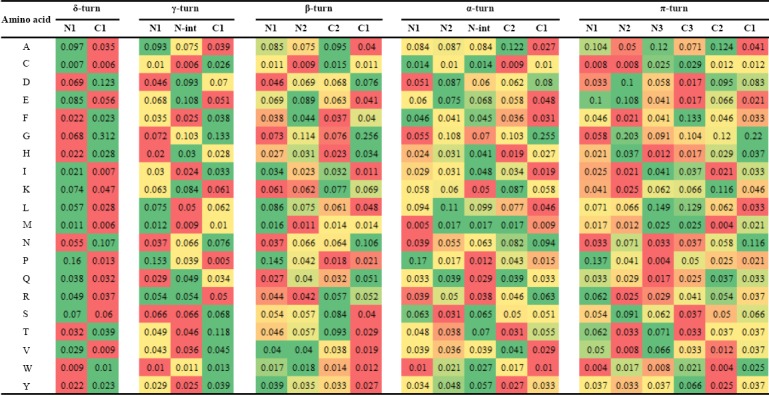
Propensities of amino acids in tight turns

This work presents the most comprehensive analysis of position-dependent properties in protein secondary structures. An exhaustive study of the frequency of occurrence of individual amino acids and physico-chemical properties was carried out on a set of 34422, 63279, and 53192 sequences corresponding to α-helix, β-strand, and tight turns, respectively. The protein sample used in this study was very large, hence unbiased, giving high confidence to the obtained results, expressed in terms of global and local propensities. Some position-dependent physico-chemical features were also studied in α-helix, β-strand, and tight turns. The amount of information collected will need a further automatic analysis in order to obtain useful predictive rules. The physicochemical properties and the data concerning their individual and pair propensities generated in this work would be crucial to start the predictive modeling. With this approach, we aimed to find some general rules that can be applied to any amino acid sequence in order to predict the stability of secondary structures.

In summary, our results suggested more compactness in N-termini of α-helix, β-strand, and tight turns secondary structures compared to C-termini. We have observed decoying periodicity in position-specific propensities of amino acids in α-helices and the length of α-helices. Meanwhile, we have shown significant differences in propensities of amino acids in different positions, which could guide the the formation of secondary structures.
